# Supplementary material for: Paracrine Signals from HIV-1 Infected Immune Cells Reprogram Cervical Cancer Pathways
Source: bioRxiv. 2025 Jun 7:2025.06.06.658239. Preprint. [Version 1] doi: 10.1101/2025.06.06.658239 (PMC12258984; doi:10.1101/2025.06.06.658239)
Supplement: 2 [file NIHPP2025.06.06.658239v1-supplement-2.pdf]

bioRxiv preprint doi: https://doi.org/10.1101/2025.06.06.658239; this version posted June 7, 2025. The copyright holder for this preprint (which was not certified by peer review) is the author/funder, who has granted bioRxiv a license to display the preprint in perpetuity. It is made available under aCC-BY 4.0 International license.

Supplementary files

Figure S1

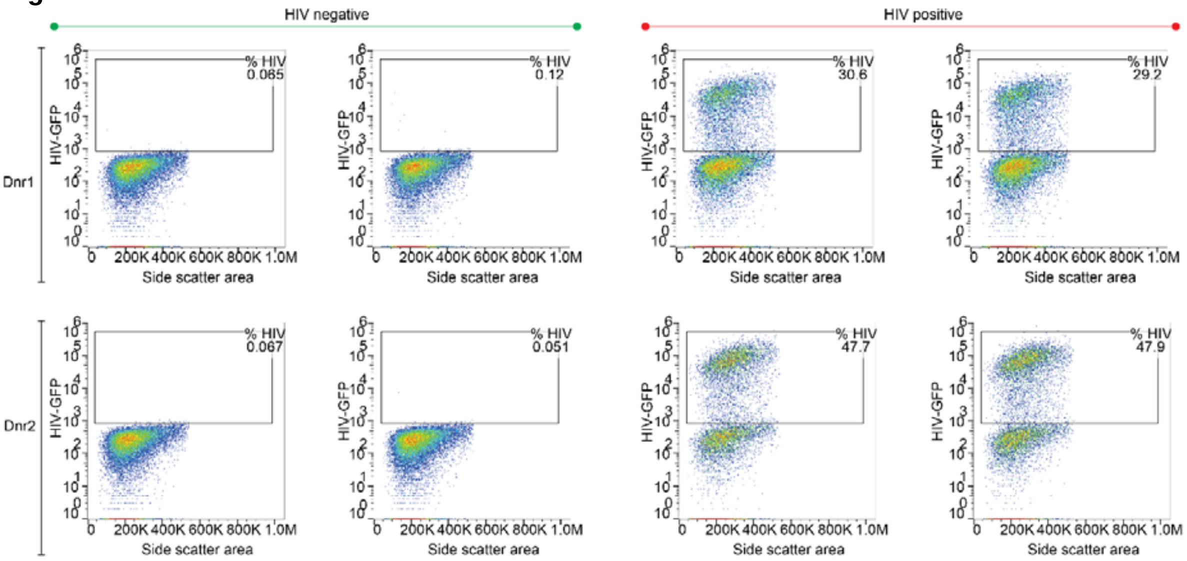

Figure S1. Infection of primary CD+ T-cells with HIV-1. Flow cytometry outputs for primary CD4+ T-cells uninfected (**Left**) or infected with a GFP-tagged replication competent HIV-1 strain (**Right**). The supernatants from these cultures were used to stimulate cervical cells.

Figure S2

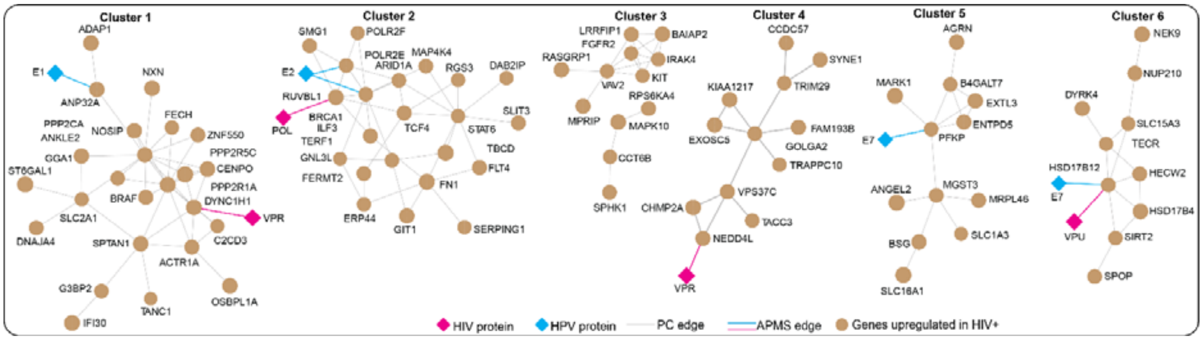

Figure S2. Network depicting the interconnection between genes with high mRNA over expression in our RNA-seq and TCGA cervical cancer datasets.

bioRxiv preprint doi: https://doi.org/10.1101/2025.06.06.658239; this version posted June 7, 2025. The copyright holder for this preprint (which was not certified by peer review) is the author/funder, who has granted bioRxiv a license to display the preprint in perpetuity. It is made available under aCC-BY 4.0 International license.

Figure S3

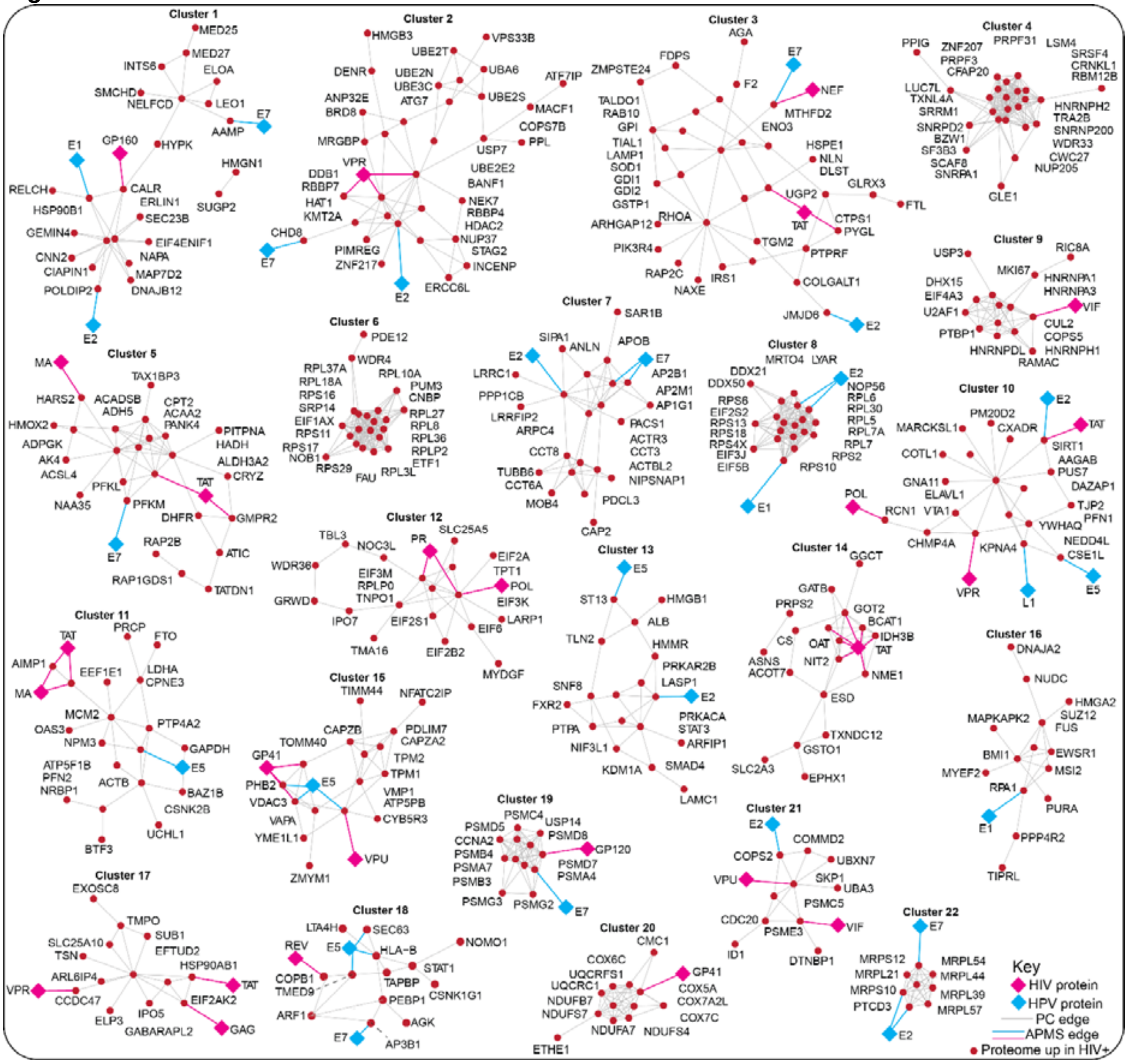

Figure S3. The network depicting interconnection between proteins upregulated in cervical cells exposed to secretome of primary CD4+ T-cells infected with HIV-1 relative to the uninfected controls.

bioRxiv preprint doi: https://doi.org/10.1101/2025.06.06.658239; this version posted June 7, 2025. The copyright holder for this preprint (which was not certified by peer review) is the author/funder, who has granted bioRxiv a license to display the preprint in perpetuity. It is made available under aCC-BY 4.0 International license.

Figure S4

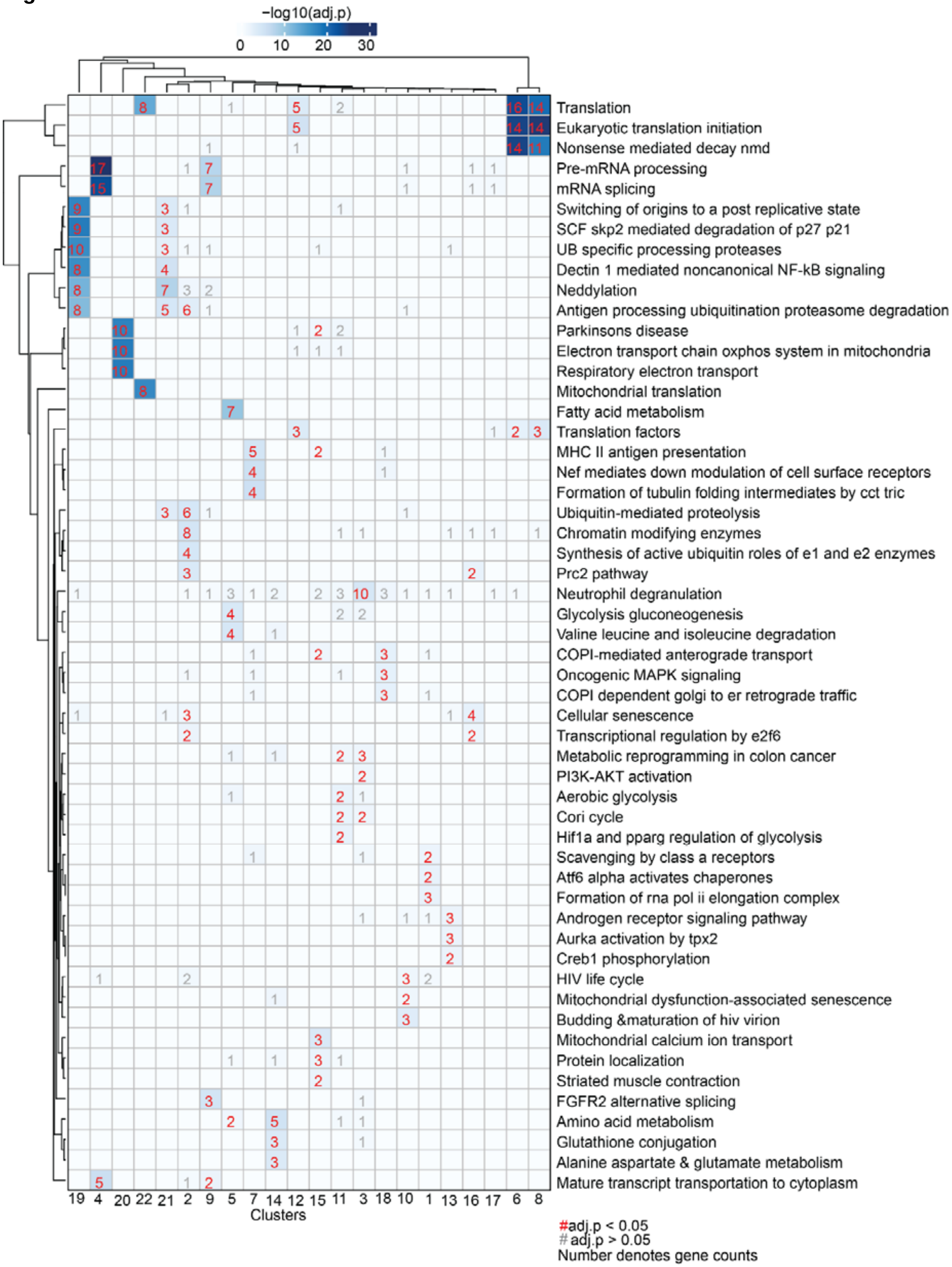

Figure S4. Gene set overrepresentation analysis of subnetworks generated from proteins upregulated in cervical cells treated with secretome of HIV-1 infected immune cells. Enrichment heatmap showing the canonical pathways associated with each of the subnetwork clusters in Figure S3. The gene set overrepresentation analysis was performed for the network subclusters. The p-values were calculated by hypergeometric test with multiple hypothesis testing correction (false discovery rate; FDR). A complete set of enriched biological pathways is provided in Table S4.

bioRxiv preprint doi: https://doi.org/10.1101/2025.06.06.658239; this version posted June 7, 2025. The copyright holder for this preprint (which was not certified by peer review) is the author/funder, who has granted bioRxiv a license to display the preprint in perpetuity. It is made available under aCC-BY 4.0 International license.

Figure S5

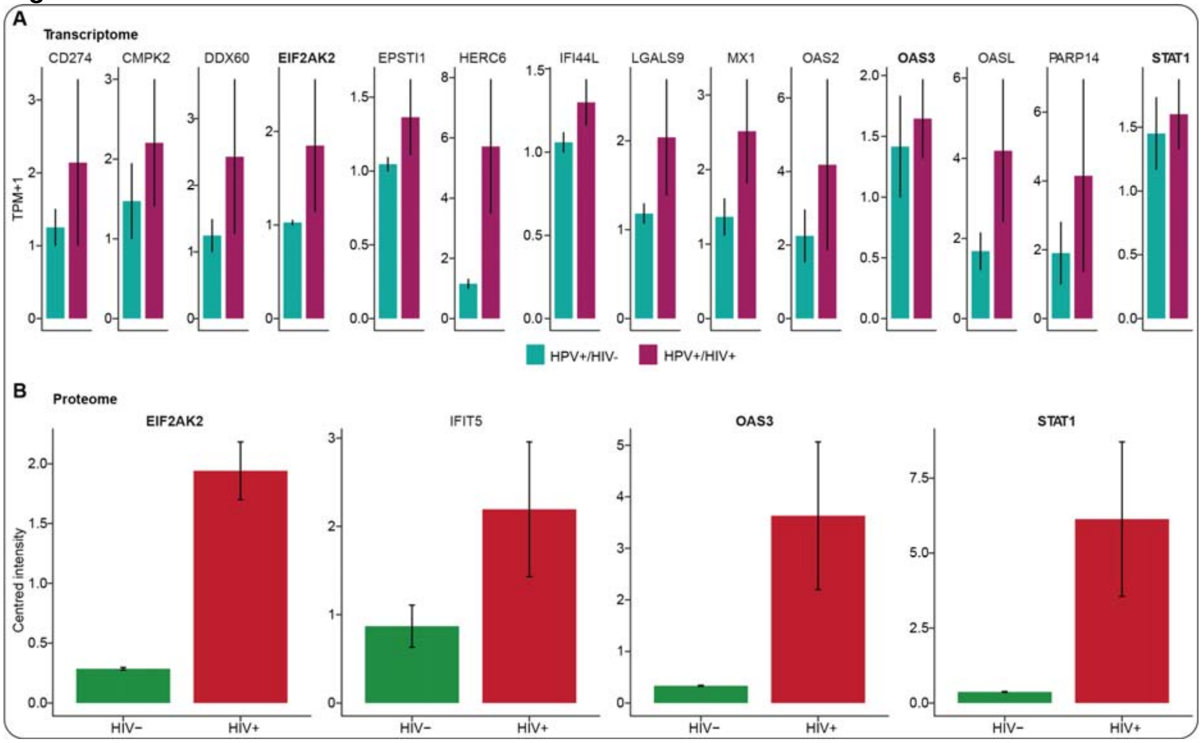

Figure S5. Comparison of the expression of ISGs in HIV-positive versus negative conditions.
(A) Bar plots displaying the normalized gene expression (*i.e.*, transcripts per million + 1) levels (mean-centered intensities) of ISGs in cervical samples of women with NC who are HIV-infected or uninfected.
(B) Box plots showing the protein expression levels (*i.e.*, mean-centered intensities) of known ISGs. The center line in each box depicts the median whereas the lower and upper edge of each box represent the 25th and 75th percentile values, respectively. The whiskers represent 1.5 times the interquartile range.
